# Thoracic spinal extradural arachnoid cyst causing Brown-Sequard-like syndrome: a rare case report and review of literature

**DOI:** 10.1093/jscr/rjad514

**Published:** 2023-09-18

**Authors:** Uchenna Ajoku, Bright Uche Nwekeala

**Affiliations:** Neurosurgery Division, Department of Surgery, University of Port Harcourt Teaching Hospital, Port Harcourt, Rivers State P.M.B. 6173, Nigeria; Neurosurgery Division, Department of Surgery, University of Port Harcourt Teaching Hospital, Port Harcourt, Rivers State P.M.B. 6173, Nigeria

**Keywords:** extradural, arachnoid, Brown-Sequard, myelopathy, magnetic resonace imaging

## Abstract

Spinal extradural arachnoid cysts are rare benign lesions occurring along the cerebrospinal axis. They may be associated with pain or varying degrees of neurological compressive symptoms. Brown-Sequard syndrome is a rare sequalae, where there is ipsilateral upper motor neuron paralysis with loss of proprioception as well as contralateral loss of pain and temperature sensation below the lesion. We present a 33-year-old female with a 6-month history of worsening right lower limb weakness and a 2-month history of right lower limb pain. Motor examination revealed right lower limb weakness as well as exaggerated knee and ankle jerk reflexes. A magnetic resonance imaging (MRI) was done, which showed an eccentrically located T4–7 cystic extradural mass causing severe cord compression. She had T4–7 laminectomies with total excision of the cyst and disconnection of the fistulous tract between the cyst and the subarachnoid space. She made full neurologic recovery with no complications.

## Introduction

Arachnoid cysts are benign cystic enlargement of the arachnoid membrane, which occur in the cerebrospinal axis. They are developmental anomalies and commonly occur in the dorsal aspect of thoracic spine, but acquired spinal arachnoid cyst (SAC) have been reported [1].

Spinal arachnoid cysts can become clinically important with sensory, motor, and/or autonomic symptoms [2–4]. Lesions causing myelopathy are usually strong indications for surgery [5–7].

Magnetic resonance imaging (MRI) is the imaging modality of choice to evaluate the anatomic location, size, and relationship with neural structures [8]. Some surgeons advocate computerised tomography scan (CT) myelography at the initial study to demonstrate any fistulous communication with the subarachnoid space not visible on MRI [9].

Brown-Sequard syndrome which is characterized by a clinical picture of hemi-section injury of the spinal cord is yet to be reported in literature from these lesions. In this clinical syndrome, there is ipsilateral upper motor neuron paralysis and loss of proprioception with contralateral loss of pain and temperature sensation. While our patient presented with right-sided lower extremity weakness and loss of proprioception, the contralateral pain and temperature loss were complete between T5 and T10 and patchy distal to this dermatomal region.

We describe this extremely rare Brown-Sequard-like presentation of thoracic spinal epidural arachnoid cyst with emphasis on the neuroanatomic basis of the clinical presentation and the nuances of management.

## Case report

A 33-year-old female with a 6-month history of worsening right lower limb weakness and a 2-month history of right lower limb pain. Further inquiry revealed that her right lower limb weakness was noticed ~15 years prior, but it had worsened in the preceding 6 months necessitating her presentation. There was no sphincteric dysfunction.

Neurologic examination revealed power of Grade 3 in L2, 3, and 4 and S1 myotomes in right lower limb, whereas in left lower limb, power was Grade 5 in L2, 3, 4, and 5 and S1 myotomes. Knee jerk and ankle jerk were exaggerated bilaterally with sustained ankle clonus. Pin-prick sensations were absent between T5 and T10 on the left and patchy distal to this. Joint position sense was also absent on the right. However, there was normal perianal sensation.

**Figure 1:**
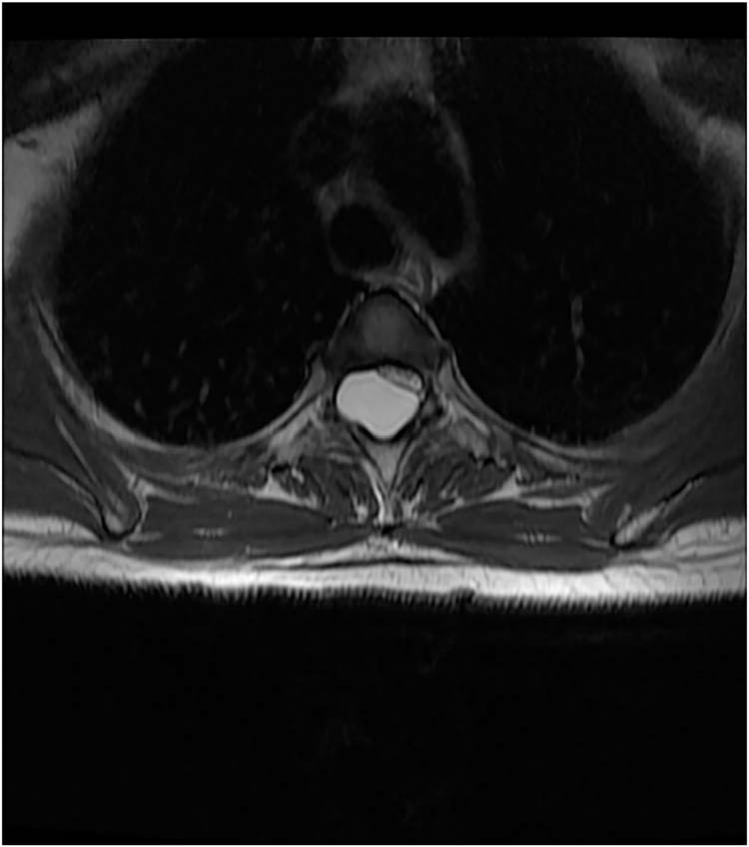
Axial T2 Image showing flattened cord displaced to the left.

**Figure 2:**
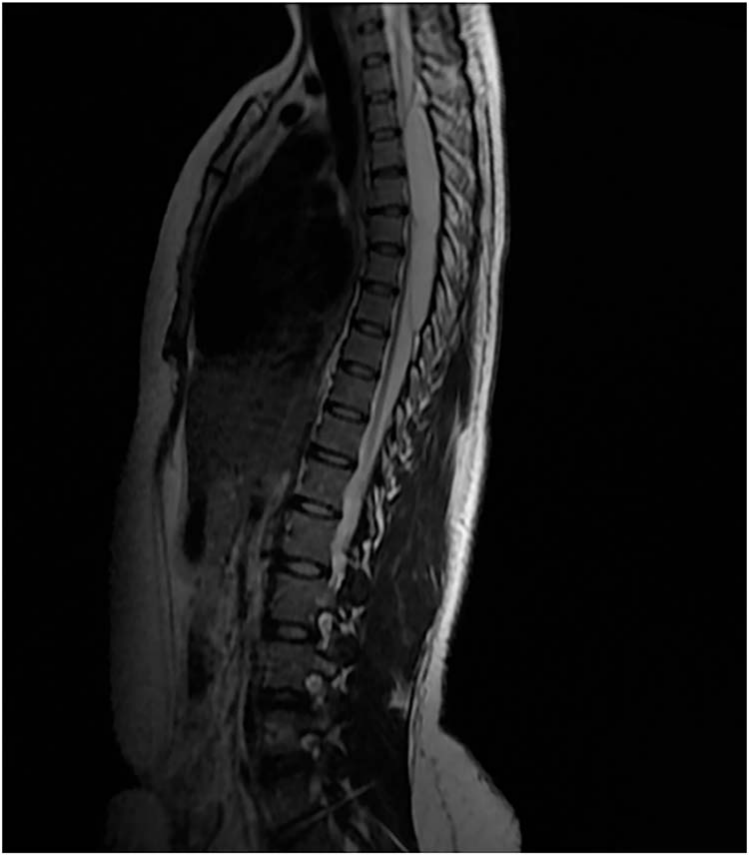
T2 sagittal showing dorsal cord compression.

An MRI of the thoracolumbar spine was done, which showed an eccentrically located T4–7 cystic extradural mass causing severe cord compression. The spinal cord was flattened and displaced to the left (Figs 1 and 2).

**Figure 3:**
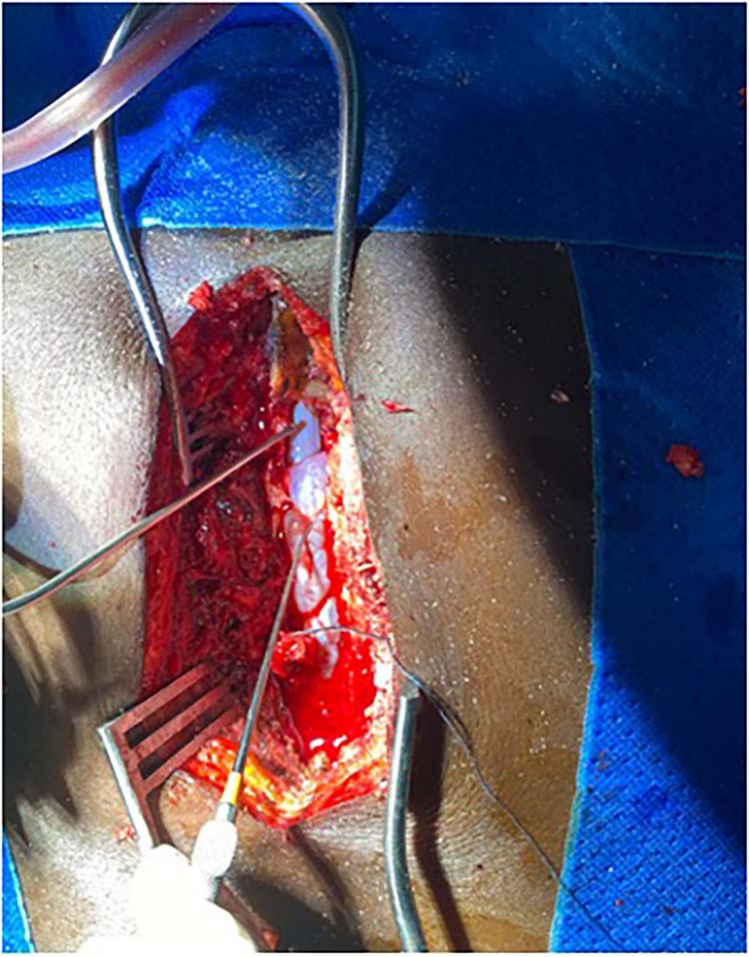
Intraop showing cyst and spinal cord.

**Figure 4:**
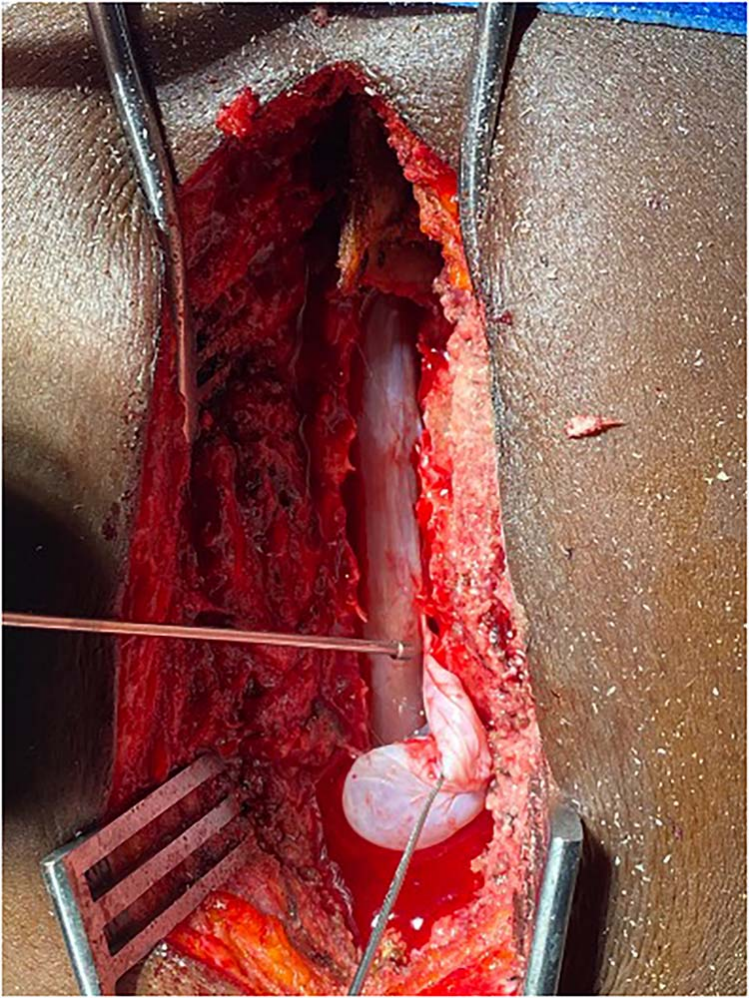
Fistulous connection of the cyst with spinal cord subarachnoid space.

**Figure 5:**
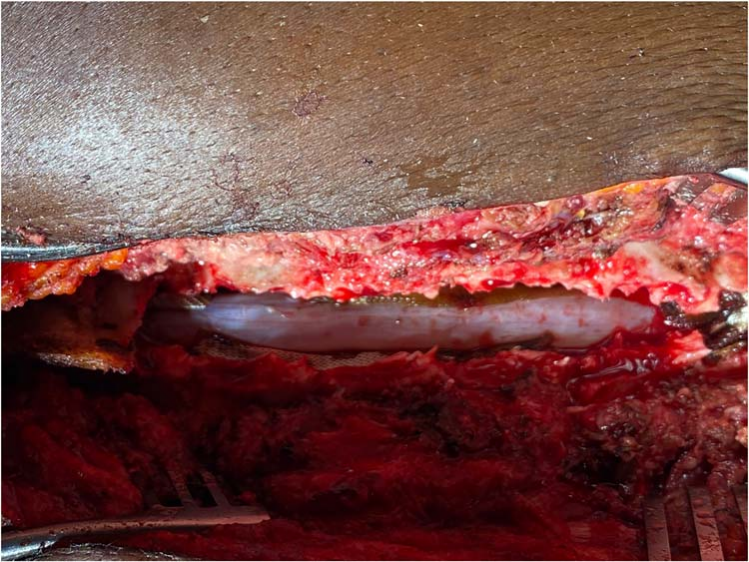
Cyst completely disconnected and excised.

Surgery was recommended with the goal of cyst excision and spinal cord decompression, and the patient gave consent for surgery under general anesthesia. She was positioned prone on an operating table, and the surgery site was prepared in a standard sterile fashion after X-ray localization. A midline incision was made to expose the surgical levels of interest. T4–7 limited laminectomies were performed, with special care taken not to violate the facet joints on both sides. Laminectomy revealed a cystic lesion lying dorsal and to the right side of the spinal cord with a fistulous connection below theT6 exiting nerve root (Figs 3 and 4). Having carefully dissected the mass off the spinal cord, the fistulous connection was then isolated, and ligated as close to the lateral edge of the dura as possible and was subsequently disconnected from the dura (Fig. 5). The surgical bed was inspected and was copiously irrigated with warm saline and the wound was closed in layers. There were no intraoperative complications.

**Figure 6:**
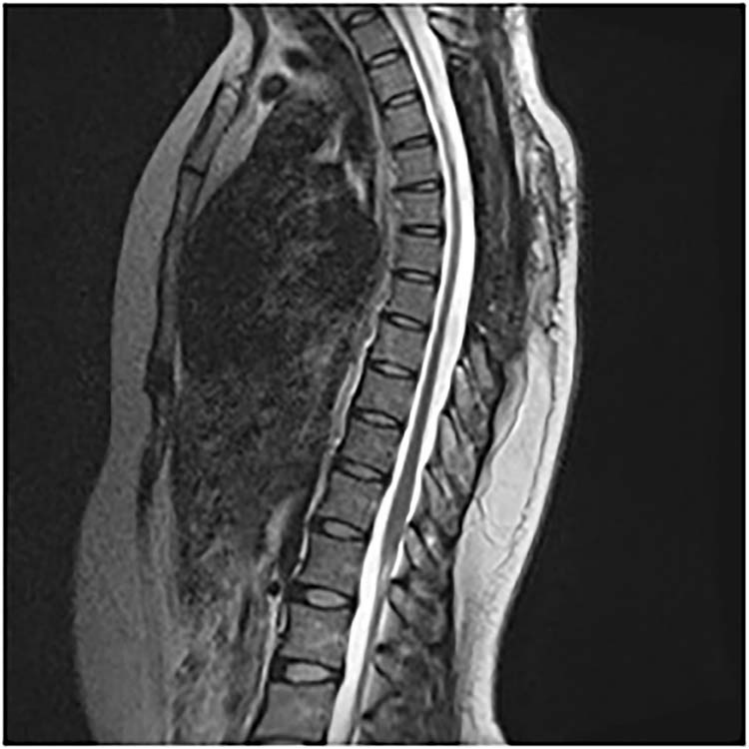
Postop sagittal.

On the first postoperative day, neurological examination revealed that power had returned to the preoperative state and her pain had significantly reduced. She was commenced on physiotherapy and on the third day postop, her limb pain had completely disappeared, while power had improved to 4 in the right lower extremity muscle groups. She was discharged on the fifth postoperative day and was to be seen in the out-patient clinic. Postop images showed total resection of cyst and gradual re-expansion of the spinal cord (Fig. 6). At 12-week follow-up, she had complete resolution of her preoperative symptoms and had returned to full functional independence.

## Discussion

Spinal extradural arachnoid cysts are rare lesions, and extensive literature search failed to reveal the neurological sequalae of Brown-Sequard syndrome. The pathophysiology of SAC involves either congenital diverticula of the dura or herniation of arachnoid through a congenital dural defect [10]. Certain individuals with SAC also have familial syndromes like Milroy’s disease comprising multiple spinal arachnoid cysts and lower-extremity lymphedema [11].

A congenital etiology is likely in this case for two reasons: the absence of a traumatic event and also the long and insidious history of over 15 years prior to her recent complaints. We hypothesize that this lesion arose from a dural defect around the right T6 nerve root, which led to the herniation of the arachnoid membrane. Similarly, the intraoperative finding of a fistulous connection between the sac and the dural inferior to the T6 exiting nerve root on the right supports the theory of dural evagination occasioned by defect at the point of T6 exiting nerve root.

Liu *et al*. gave a pictorial illustration of the fluid dynamics involved in the propagation and enlargement of SAC once formed [10]. Also, the “ball-valve” mechanism in the communicating pedicle suggests that pulsatile CSF dynamics result in cyst expansion by causing flow of CSF from the intradural space into the cyst through pressure differential created by the intermittent surges of CSF pressure [3].

The clinical manifestation of SAC would depend on the site and degree of spinal cord and/or nerve root compression [1]. In our patient, the cyst was located dorsal and to the right hemi-cord, which explains the Brown-Sequard syndrome manifestation. The mainstay of treatment in patients with neurological deficits is complete surgical excision [12]. The surgical nuance lies in identifying the fistulous connections intraoperatively and disconnecting it from the dura. Percutaneous procedures like needle aspiration, needle fenestration have been tried with varying results, and open procedures like surgical resection, fenestration, and cyst shunting appear to give the best results [5].

## Conclusion

Spinal arachnoid cyst is a rare clinical entity. Brown-Sequard syndrome because of SAC is yet to be reported in literature. Timely and meticulous surgical intervention ensuring that any fistulous communication with the intradural space is disconnected is required for good clinical outcome.

## Data Availability

Data sharing is available from the authors upon reasonable request and with the patient’s permission.
